# Hyperion: A New Computational Tool for Relativistic Ab Initio Hyperfine Coupling

**DOI:** 10.1021/acs.jctc.2c00257

**Published:** 2022-07-01

**Authors:** Letitia Birnoschi, Nicholas F. Chilton

**Affiliations:** Department of Chemistry, The University of Manchester, Oxford Road, Manchester, M13 9PL, United Kingdom

## Abstract

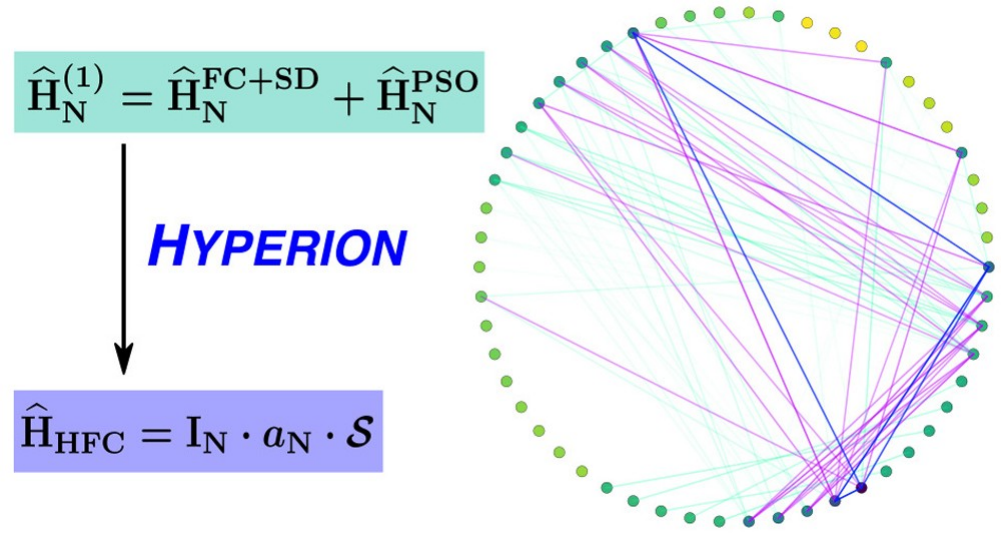

Herein we describe Hyperion, a new program for computing relativistic picture-change-corrected magnetic resonance parameters from scalar relativistic active space wave functions, with or without spin–orbit coupling (SOC) included *a posteriori*. Hyperion also includes a new orbital decomposition method for assisting active space selection for calculations of hyperfine coupling. For benchmarking purposes, we determine hyperfine coupling constants of selected alkali metal, transition metal, and lanthanide atoms, based on complete active space self-consistent field spin–orbit calculations in OpenMolcas. Our results are in excellent agreement with experimental data from atomic spectroscopy as well as theoretical predictions from four-component relativistic calculations.

## 1 Introduction

Magnetic resonance spectroscopy techniques, such as electron paramagnetic resonance (EPR) and nuclear magnetic resonance (NMR), are capable of providing very accurate information on the interactions between electron spins, nuclear spins, and external magnetic fields. The information available from EPR and NMR spectra is often encoded via iterative fitting of the experimental data to a model spin Hamiltonian in a set of effective parameters, each related to a specific type of coupling between magnetic entities. Quantities probing interactions between electron and nuclear spins, namely, EPR hyperfine coupling constants (HFCCs) and paramagnetic NMR shifts, depend strongly on unpaired electron (spin) density^1,2^ and are central to the study of various phenomena encountered in molecular systems of interest. Examples include using HFCCs as proxies for covalency in actinide complexes,^3^ quantifying spin decoherence in molecular qubits via theoretically determined hyperfine coupling (HFC) tensors,^4^ and employing contact NMR shifts—which have an intrinsic dependence on HFC parameters^5^—to understand the solution structure and magnetic properties of paramagnetic MRI contrast agents.^6^

The steady evolution of electronic structure algorithms has made it computationally feasible to study heavy-element complexes fully *ab initio*. As the atomic number increases, relativistic effects become more important, to the extent where they cannot be regarded as mere perturbations of the Schrödinger picture; instead, a four-component Dirac formalism becomes necessary. The onset of the relativistic regime is especially important for magnetic interactions, which couple the electronic and positronic degrees of freedom of a Dirac spinor, therefore requiring an explicit description of the latter. Hence, theoretical frameworks developed for magnetic properties such as HFC must account for relativistic effects—both spin-independent (scalar relativistic, SR) and spin-dependent (e.g., spin–orbit coupling, SOC)—to ensure a wide range of applicability and to keep up with the latest experimental advances.

HFC between an unpaired electron spin **S** and a nuclear spin **I**_N_ is most frequently modeled non-relativistically as the sum of anisotropic dipolar coupling, known as the spin-dipole (SD) mechanism, and isotropic Fermi coupling (FC)^7^
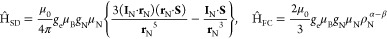
1where we use μ_0_, *g*_e_, μ_B_, *g*_N_, μ_N_, and **I**_N_ to denote the vacuum permeability, electron *g*-factor, Bohr magneton, nuclear *g*-factor, nuclear magneton, and nuclear spin vector, respectively; **r**_N_ = **r** – **R**_N_ is the position vector of the unpaired electron with respect to the magnetic nucleus. The strength of FC is proportional to the spin population at the magnetic nucleus, ρ_N_^α–β^, thus providing a convenient probe for spin delocalization in a molecule. Although the single-configurational view of electronic structure suggests that this term arises solely through s-type atomic orbital (AO) contributions to the singly occupied molecular orbital (SOMO), several factors complicate this interpretation.

Differences between the interaction of core spin-up and spin-down electrons with the unpaired spin give rise to *spin polarization* (SP),^2^ which gives additional contributions to the isotropic HFC and cannot be described by a single electronic configuration. Thus, the quantitative interpretation of HFCCs requires quantum chemical techniques to model the electronic structure accurately and capture SP effects. For this purpose, there are two possible solutions: spin-unrestricted single-configurational methods, such as unrestricted Hartree–Fock (UHF) and unrestricted Kohn–Sham density functional theory (UKS-DFT), which offer low computational cost at the expense of broken spin symmetry, and more expensive spin-adapted multiconfigurational methods, which preserve spin symmetry and are designed to handle electron correlation.

Unrestricted DFT is currently the most widely used approach for determining HFCCs, due to the lower computational cost relative to wave-function-based (*ab initio*) algorithms. Hybrid functionals give good predictions for HFCCs of organic radicals and transition metal complexes;^8,9^ however, the accuracy of these results is believed to be caused by fortuitous error cancellation.^10^ Moreover, a recent study^11^ shows that the best choice of functional for HFC is system-dependent. Aside from HFC-related shortcomings, the single-configurational framework underlying Kohn–Sham DFT is inappropriate for describing static correlation, an important feature of f-element complexes. Such systems require a multiconfigurational approach, usually in the form of active space wave function optimization techniques such as complete active space self-consistent field (CASSCF), in order to obtain meaningful predictions of energies and molecular properties.

Although far from black-box, active space algorithms are not only cheaper than fully correlated alternatives such as full configuration interaction or coupled cluster methods but also more flexible, as the active space is user-defined. This framework can be leveraged to obtain accurate theoretical HFCCs by including, in addition to static correlation, a selection of dynamical correlation effects (e.g., SP) that significantly influence HFC. The challenge, then, is developing a strategy for choosing computationally feasible active spaces that result in accurate theoretical HFCCs. Previous studies employing multireference configuration interaction (MR-CI) algorithms have already analyzed the convergence of HFCCs with respect to CI excitation level, orbital selection threshold, and basis set completeness.^2,12−19^ However, most of the established trends are only applicable to the non-relativistic regime; in particular, the observations about AO contributions to HFCCs result directly from the delta distribution form of the FC term (eq 1, right), which is specific to the non-relativistic Schrödinger–Pauli framework.^20^ Relativistic treatments of HFC hence require new work to establish updated guidelines for theoretical investigations.

Fully relativistic four-component approaches are unfeasible for all but the simplest systems,^21,22^ unless combined with a low-cost electronic structure algorithm such as Dirac–Hartree–Fock (DHF)^23^ or DFT.^24^ As such, multiple strategies that decouple the upper (electronic) and lower (positronic) components of the Dirac Hamiltonian have been developed to lower the cost of relativistic calculations for application to real molecular systems. Use of a decoupling transformation is, in effect, a change in the reference frame of the wave function, and therefore, it must also be applied to the property operators; ignoring this second step leads to the so-called picture-change error (PCE).^25^ Due to the picture-change correction, relativistic property operators are different from their non-relativistic counterparts and, in the case of relativistic HFC, the isotropic contribution is no longer proportional to the spin density at the nucleus.

Approximate (quasi-relativistic) decoupling techniques, such as the regular approximation (RA) and Douglas–Kroll–Hess (DKH), are now fairly widespread and provide excellent predictions for the energies of most relativistic systems.^26,27^ These two-component approaches can be simplified further by disregarding spin-dependent effects, yielding a one-component scalar relativistic formalism. For magnetic resonance applications, the zeroth-order regular approximation (ZORA) is widely used in combination with DFT.^28−31^ Despite providing a good description of valence properties,^32,33^ ZORA affords large errors for core-dependent properties of heavy-element systems (e.g., core-hole X-ray excitations or absolute nuclear shielding tensors);^32,34^ DKH is significantly more accurate in this respect. Indeed, HFCCs obtained using SR-DKH2 operators are in good agreement with experiment,^35,36^ although Nguyen Lan et al.^37^ show that a higher-order decoupling transformation (DKH3) is needed for a converged relativistic HFC picture. Quasi-relativistic HFCCs can also be derived via the infinite-order regular approximation (IORA), and when combined with a multiconfigurational wave function method, this approach produces highly accurate atomic hyperfine structure constants for alkali and coinage metals.^38^

Exact two-component decoupling schemes are also available, together with one-component SR variants. For the purpose of theoretical HFC, these offer two main improvements over quasi-relativistic theories: there is no uncertainty regarding the appropriate order of decoupling, and picture-change corrections are straightforward to implement via matrix multiplication. The exact-2-component (X2C)^39−41^ and normalized elimination of the small component (NESC)^42^ approaches are two popular, fully numerical, choices for theoretical studies of relativistic HFC. In such cases, it is worth keeping in mind that the form of the relativistic HFC operator changes on a case-by-case basis, since the picture-change transformation is only defined in matrix form.

Our goal was to devise a general methodology for determining relativistic, picture-change-corrected HFCCs for chemical systems of arbitrary size and complexity. With this in mind, we developed Hyperion, a Python-based program that computes SR-X2C-decoupled magnetic resonance parameters from complete active space (CAS) or restricted active space (RAS) wave functions, with or without spin–orbit coupling (SOC) added *a posteriori* (CASSCF-SO/RASSCF-SO); a method based on the same theoretical formalism has been developed in parallel by Autschbach and co-workers and implemented as part of OpenMolcas.^41^ Although our code has to date been tested only with OpenMolcas, Hyperion is a stand-alone package that can be straightforwardly extended to allow inputs from other quantum chemical software. Herein, we demonstrate the use of Hyperion to obtain HFCCs of selected atoms and benchmark our results against experimental data from atomic spectroscopy and predictions from four-component calculations. As well as showing the performance of Hyperion, we demonstrate various strategies for tackling specific combinations of electron correlation, SR, and SOC effects, highlighting both merits and limitations of CASSCF-SO/RASSCF-SO methods. The comparatively small number of electrons and high symmetry of atoms mean that high levels of theory are achievable with relatively little computational cost, but as our goal is to extend our approach to molecules in the near future, we work with usual electronic structure approximations, such as contracted basis sets and RAS subspaces of limited size, which are the only feasible strategies for molecular calculations.

## 2 Theory

### 2.1 SR-X2C Magnetic Properties

Here, operators are denoted using the hat symbol (T̂) and vector quantities are shown in bold (**A**). One-component and two-component operators are represented using normal font (Ĥ_SF-X2C,UU_^(1)^), while bold-italic notation (***H***_SF-X2C,UU_^(1)^) is used for their matrix representations in a scalar AO basis. We denote four-component operators as matrix operators (**Ĥ**_mDE_) to emphasize their 2 × 2 block structure. Finally, we employ double-struck notation () for matrix representations of four-component operators.

The starting point for any relativistic treatment of magnetic properties is the four-component Dirac equation under a scalar potential *V* and a vector potential **A**
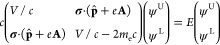
2where **σ** is the 3-vector of Pauli spin matrices, **p̂** is the electron linear momentum operator, *m*_e_ is the electron mass, and *c* is the speed of light in a vacuum; SI units are used throughout this work. We represent all four-component operators in block form, with each matrix element denoting a two-component operator. Note that, in two-component equations, **σ** = 2**s**, where **s** is the electron spin vector.

The Dirac wave function, also known as a four-component spinor, has an upper component ψ^U^, sometimes referred to as the large component, as well as a lower component (or small component) ψ^L^. In order for eq 2 to approach the correct non-relativistic limit, the basis sets chosen for ψ^U^ and ψ^L^ must obey the restricted kinetic balance (RKB) condition,^43−46^ namely, that, for an upper component basis set {ϕ_μ_}, the lower component basis set is . Although using RKB alone is formally justified only in the absence of magnetic fields, this approach is reasonable, as the magnetic-field-dependent terms are treated as a perturbation herein (*vide infra*). By substituting the RKB condition into the four-component Hamiltonian, we arrive at the modified Dirac equation^47,48^
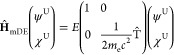
3with
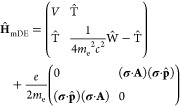
4
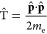
5
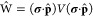
6Notice that the lower component of eq 2 has been replaced by the pseudo-upper component χ^U^, which can be represented in the same basis as that used for ψ^U^. Additionally, the modified Dirac Hamiltonian (eq 4) has been separated into **A**-independent and **A**-dependent contributions; we henceforth treat the **A**-dependent term as a first-order perturbation, **Ĥ**^(1)^. Using the Dirac relation, we rewrite Ŵ as
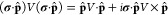
7and discard the **σ**-dependent term to obtain a spin-free four-component operator. By ignoring the spin-dependent contribution, this relativistic formalism becomes compatible with the one-component framework employed by most electronic structure packages, including OpenMolcas. The resulting spin-free (SF) modified Dirac operator is
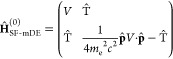
8which shall serve as the zeroth-order (unperturbed) Hamiltonian within our theoretical framework.

Scalar relativistic exact-2-component (SR-X2C) theory^49−52^ can be employed to determine a unitary transformation matrix  (eq 9) that decouples the upper and pseudo-upper components by block-diagonalizing the matrix representation of **Ĥ**_SF-mDE_^(0)^, herein denoted as . The electronic problem can then be solved by diagonalization of the upper–upper matrix, ***H***_SF-X2C,UU_^(0)^, without any reference to the other two-component blocks.
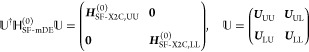
9The SF-X2C-decoupled perturbation is obtained in an analogous manner, by applying the same unitary transformation to . For simplicity, we employ operator notation throughout the remainder of this section; however, it should be noted that, within the Hyperion implementation, the decoupling transformation of **Ĥ**^(1)^ and indeed all subsequent steps in the calculation of expectation values are carried out in matrix form. As this work pertains to electronic properties, only the upper–upper block of the transformed operator
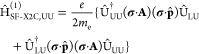
10is of interest, and we henceforth drop the UU subscript from Hamiltonian operator notation.

The interaction between an unpaired electron and a nuclear spin, i.e., HFC, can be derived from eq 10 by substituting in the vector potential **A**_N_ induced by a point-like magnetic dipole N
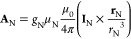
11Alternatively, the nuclear magnetic dipole can be modeled as a Gaussian distribution (eqs 12 and 13)—this is also implemented in Hyperion; however, results herein employ the point nucleus expression for simplicity.
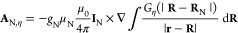
12
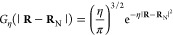
13The electronic HFC perturbation operator within the SF-X2C framework is therefore
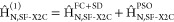
14
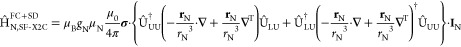
15
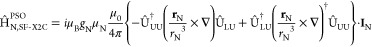
16where we distinguish between a spin-dependent contribution, Ĥ_N,SF-X2C_^FC+SD^, and an imaginary, spin-independent contribution, Ĥ_N,SF-X2C_^PSO^. The former reduces to the sum of the FC and SD operators in the non-relativistic limit, while the latter represents the interaction between the electronic orbital angular momentum and the nuclear spin and is known as the paramagnetic spin–orbit coupling (PSO) term.

It is worth highlighting that eq 15 does not include a delta-function, that would imply only contributions at the nucleus and characteristic of traditional interpretations of FC; as shown by Kutzelnigg,^20^ this hallmark only arises in the non-relativistic limit of a two-component framework. As a result, relativistic spin-dependent HFC cannot be interpreted as a combination of classical dipolar coupling (SD term) and an FC term sampling the spin density at nucleus N.^7^

An important aspect that bears discussion is the spin–orbit contribution to HFC. Herein, SOC effects are included in the zeroth-order Hamiltonian, and as such, the spin–orbit HFC contribution is a first-order property,^34^ computed as the expectation of the PSO operator, eq 16. An alternative approach involves modeling SOC as a perturbation and mapping the HFC operator onto a true spin effective Hamiltonian,;^53^ in this case, the spin–orbit HFC term only appears in the second-order response, and the second-order HFC energy includes both the PSO interaction and SOC effects on the wave function.^54^ Therefore, even though both approaches yield spin–orbit contributions to the HFC energy, the two are not directly comparable. The perturbative SOC formalism is the *de facto* choice in non-relativistic HFC studies;^9,31^ meanwhile, relativistic four- and two-component methods already account for SOC in the wave function; hence, the HFC energy includes a first-order PSO term.^55^ Within a one-component formalism such as that employed by OpenMolcas and Hyperion, SOC can be modeled using either approach. Nevertheless, as we are targeting a variety of chemical systems, including atoms and molecules where strong SOC significantly alters the manifold of electronic states, we do not implement the perturbative SOC method within Hyperion. Instead, effects of SOC are included *a posteriori* in the zeroth order wave function via the restricted active space state interaction (RASSI) formalism^56^ before calculation of the hyperfine coupling.

### 2.2 Spin Hamiltonian Parametrizations

The cornerstone of EPR theory is the spin Hamiltonian, an effective operator describing all of the relevant magnetic interactions as couplings in a (2*S* + 1)-dimensional space, where *S* is the spin quantum number of the system. The HFC spin Hamiltonian is
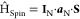
17where the HFC tensor ***a***_N_ quantifies the strength and anisotropy of the interaction. Quantum mechanical determinations of EPR parameters are based on mapping *ab initio* operators onto the spin Hamiltonian and differentiating to obtain the tensor components of ***a***_N_:
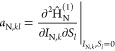
18where . In a spin-free/scalar relativistic framework, provided the electronic system is spin-only (no SOC), the tensor components are computed as
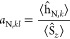
19where we have defined the vector operator **ĥ**_N_ as
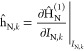
20Note that in the case of spin-free electronic structure approaches, such as spin-free CASSCF, the spin projection expectation value ⟨Ŝ_*z*_⟩ is exactly equal to *S*.

This spin-only parametrization is not appropriate, however, for systems exhibiting non-negligible SOC, as *S* is no longer a good quantum number. Instead, the size of the model space is chosen as the number of low-lying electronic states in the SO-coupled energy spectrum, resulting in an effective  multiplet, where  is the pseudospin quantum number. Replacing *S* with  in eq 17 yields the pseudospin Hamiltonian, from which the elements of symmetrized tensor ***a***_N_***a***_N_^T^ are computed via the method of Chibotaru^57^
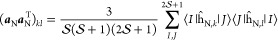
21where |*I*⟩ and |*J*⟩ denote eigenstates in the pseudospin manifold. A similar approach is widely used to derive g-tensors for strongly SO-coupled systems.^58^ The eigenvectors and eigenvalues of the symmetrized tensors correspond to the principal axes and the squared principal values of the original tensors; as a result, the pseudospin parametrization yields *unsigned* HFCCs. Although the present work is focused on HFCC magnitude, we note in passing that sign information becomes important if the parameters determined theoretically are employed in further computational work, such as simulations of EPR spectra.

Finally, Hyperion is also capable of determining SF-X2C g-values, using a similar methodology to that described above for HFCCs. The key expressions employed in g-value calculations can be easily obtained by replacing eqs 11 and 17 with the vector potential induced by an external magnetic field and with the electron-Zeeman spin Hamiltonian, respectively. This additional capability will be assessed as part of a future study on molecules.

### 2.3 HFC Orbital Decomposition

One aspect of theoretical determination of HFCCs using multiconfigurational methods is choosing the most appropriate active space. This is largely a task of trial and error, for which it is hard to define general rules. Hence, we have developed an orbital decomposition method to assess the involvement of particular MOs in the spin-dependent HFCC of a spin-free state Ψ^SS^. To do so, we use the second quantization formalism to represent the expectation value of each vector operator component ĥ_N,*k*_^FC+SD^ as a sum over pairs of MOs {*μν*}
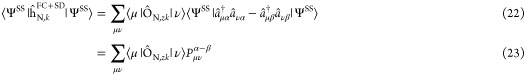
22where *â*^†^ and *â* denote spin–orbital creation and annihilation operators, respectively, and *P*_*μν*_^α–β^ is a spin density matrix element. We have used Ô_N,*zk*_ to denote the *l* = *z* components of a rank-2 tensor operator defined as
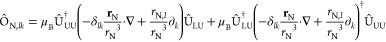
24Note that **Ô**_N_ represents the spatial part of **h**_N,SF-X2C_^FC+SD^, as derived via eqs 15 and 20.

We consider each term from the summation in eq 23 separately, combine the *k* = *x*, *y*, *z* components of **Ô**_N,*z*_ via the vector norm, and divide the result by the spin projection to obtain a two-dimensional symmetric matrix  with elements
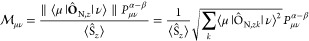
25The  matrix contains the same information as the spin-dependent HFCC, albeit in a modified form wherein MO degrees of freedom are not integrated out. We note that the factor of ⟨Ŝ_*z*_⟩^–1^, which reduces to *S*^–1^ for spin-free states, mimics the expression for HFC operators in the spin-only formalism. Within this representation, the diagonal elements  can be interpreted as individual orbital contributions, while the coupling between two different orbitals is quantified by .

**Figure 1:**
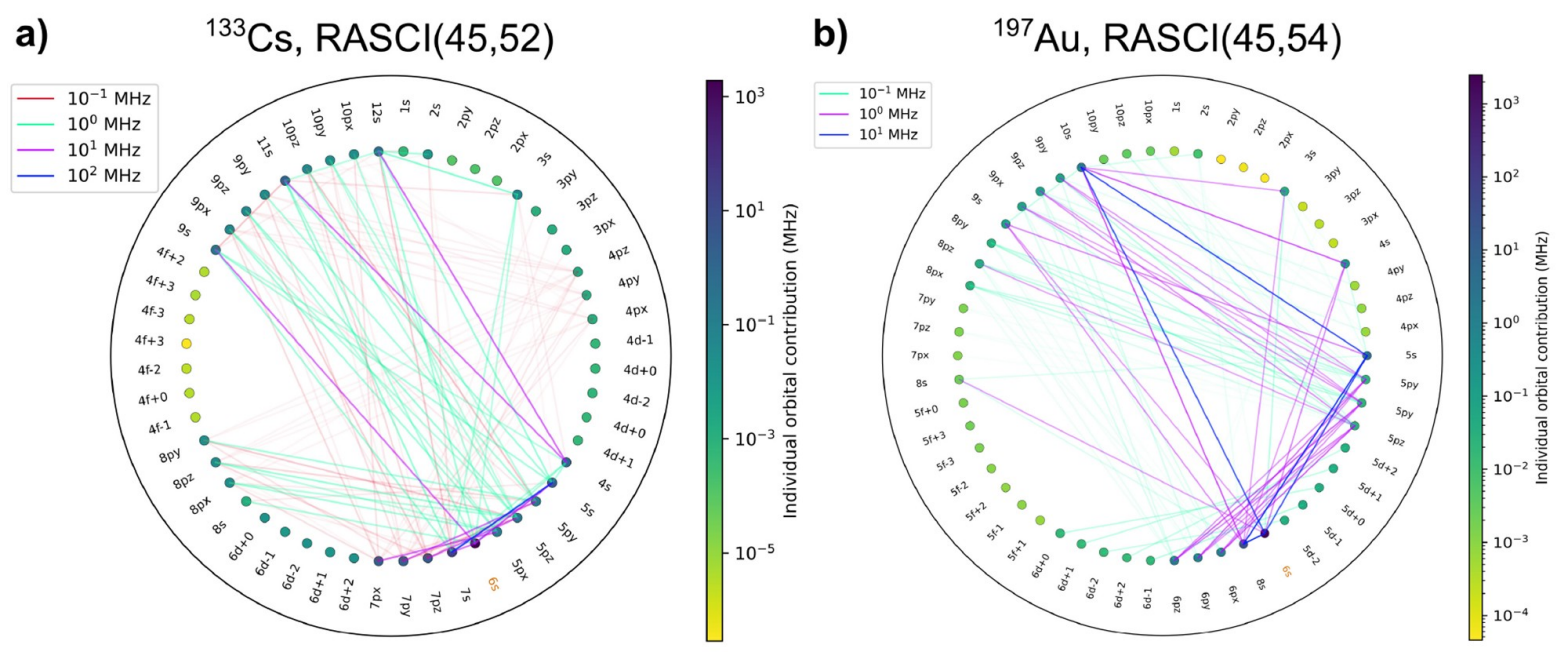
HFC orbital decomposition diagrams obtained from relativistic determinations of HFCCs using Hyperion. (a) ^133^Cs atom, RASCI(45,52) using CASSCF(11,15)-optimized orbitals; (b) ^197^Au atom, RASCI(45,54) using RASSCF(19,26)-optimized orbitals. Lines across the diagram correspond to off-diagonal matrix elements of . Individual orbital contributions, as indicated by circular markers, correspond to diagonal matrix elements .

Inspired by density matrix renormalization group (DMRG) entanglement diagrams,^59^ we designed a similar pictorial representation of orbital involvement in HFC (Figure 1), using the matrix elements of . Individual orbital contributions are shown as markers on the circumference of a circle, while pairwise contributions are shown as chords, color-coded by order of magnitude.

It is worth emphasizing that this model is based on a CI-type wave function that is not relaxed after applying the first-order HFC perturbation and that our orbital decomposition diagrams only show a static picture of HFC. To further understand this, we adopt the *direct/indirect* terminology used by Engels^14^ to describe the influence of specific configurations in a CI wave function. The HFC orbital decomposition matrix  for a given electronic state is computed via its spin density matrix, which is completely determined by the CI expansion. The *direct* influence of an (active) orbital or pair of orbitals, as indicated by , is then proportional to the total CI contribution from all configurations where the orbitals of interest are singly occupied. However, the spin density associated with one orbital can vary between different active space selections, due to the *indirect* effect of all of the other correlated orbitals. Therefore, the active space selection should not be based solely on the orbital decomposition diagram. HFC calculations should be carried out both with and without a specific orbital in the CAS/RAS to determine the magnitude of its indirect effect.

## 3 Computational Details

All electronic structure calculations use a local version of OpenMolcas^60^ v19.11 adapted to print the X2C decoupling matrices, while the spatial HFC integrals are evaluated analytically with Libcint.^61^ Full ANO-RCC basis sets^62−66^ are used throughout to ensure sufficient flexibility and to maintain consistency with previous relativistic HFC studies.^39,41,67^ The electronic wave function is optimized using either the complete active space self-consistent field (CASSCF) approach^68^ or a restricted active space (RAS) approach^69^ with (RASSCF) or without orbital optimization (RASCI). For RASSCF/RASCI calculations, the maximum allowed number of RAS1 holes and the maximum allowed number of RAS3 electrons are both set to 2. Spherical symmetry in the optimized MOs is enforced via the ATOM keyword, which prevents rotations among orbitals belonging to different representations of the full rotation group. Where necessary, SOC is added *a posteriori* using the RASSI approach,^56^ which employs a Pauli spin–orbit Hamiltonian,^70^ represented using atomic mean field integrals (AMFIs).^71^ To keep RAS calculations tractable, the RAS2 subspace is restricted to the partially occupied shells. As such, for ^2^S_1/2_ systems, only the singly occupied s orbital is included in RAS2, while, for transition metals or lanthanides with partially filled d or f shells, respectively, RAS2 is made up of the valence *n*d or *n*f and (*n* + 1)s orbitals as required.

## 4 Results and Discussion

### 4.1 Spin Polarization vs Electron Correlation

We take this opportunity to discuss a conceptual aspect that is often encountered in computational HFC literature: the overlap between spin polarization and electron correlation. Both effects have physical interpretations related to the interaction between electrons in a many-electron system; however, a distinction arises in electronic structure theory due to the use of single-configurational (also known as mean-field) references with respect to which perturbations such as SP, static correlation, and dynamic correlation are described.

Unrestricted Hartree–Fock (UHF) theory is the simplest wave-function-based electronic structure framework that accounts for SP, albeit not accurately, due to spin contamination. The UHF wave function can be expressed as a perturbation expansion from a restricted open-shell Hartree–Fock (ROHF) reference, with the first-order term comprising only single excitations.^72^ It can be deduced that SP arises mainly from singly excited configurations; a spin-adapted CI ansatz with singles (S–CI) should therefore be able to provide a more accurate, non-spin-contaminated description of SP.^2^

Electron correlation—the instantaneous interaction of electrons, which is not captured by a mean-field ansatz—is also routinely described by a CI wave function, which is most accurate when all possible configurations obtained by distributing all electrons among the available orbitals are included (full CI, FCI). The correlation energy is defined as the difference between the FCI and the reference Hartree–Fock energies. For open-shell systems represented in a spin-adapted framework (i.e., with a ROHF reference), it follows that electron correlation includes an SP contribution,^72^ whose magnitude directly relates to the overall weighting of single excitations in the CI expansion. Note, however, that, if a UHF reference is used instead, the boundary between SP and “other” correlation becomes unclear. This is fortunately not a concern herein, as we employ spin-adapted wave function methods exclusively.

In a comprehensive review of the spin-polarization model for HFC, Chipman^2^ refers to *second- and higher-order correlation effects*/*true correlation*, thus dividing spin-polarizing single excitations from other excited configurations sampled by FCI. While this split view helps balance computational efficiency with accuracy in multiconfigurational calculations, it has also led to a prevalence in the HFC literature of single-excitation-only results, with no discussion of higher-order effects.^41,67^ This is likely a safe approximation for systems such as simple organic radicals;^73^ however, there is no reason to assume *a priori* that the SP contribution to HFC is more important than contributions from higher-order excitations. In fact, early work shows that high-level CI approximations are required even for second period atoms to achieve quantitative agreement with experiment.^16^ For the atomic systems studied here, we attempt to include most correlation afforded by a chosen active space using either CASSCF or, where this is unfeasible, RASSCF/RASCI with single and double excitations.

### 4.2 Alkali Metals

We first apply our methodology to study the hyperfine structure of atoms in the alkali series. This is a popular test set for theoretical HFC computations, requiring a good description of electron correlation, as well as an appropriate treatment of relativistic effects for the heavier elements.^22,74,75^Table 1 shows predicted (unsigned) HFCCs for the ^2^S_1/2_ ground state of each alkali atom, determined via active space electronic structure methods. We note that our CASSCF(1,1) results are very close to the four-component Hartree–Fock data reported by Talukdar,^22^ suggesting that, at least for the direct contribution to HFC, relativistic effects are correctly accounted for with the X2C decoupling in Hyperion. In order to include correlation contributions to the spin density, the active space must be expanded beyond the SOMO; the CAS size is limited to a maximum of 18 orbitals, and we turn to RAS methods to explore electron correlation effects in a larger orbital space. Note that, due to the computational scaling of RAS algorithms, we restrict the RAS2 subspace to the SOMO. HFCCs converge the fastest when the CASSCF algorithm is used, with CASSCF(11,15) results within approximately 10% of experimental HFC data for alkali Na–Fr. This contrasts with RASSCF and RASCI results, which display slower convergence with active space size and only reach CASSCF accuracy when the majority of MOs are correlated.

**Table 1:** Unsigned Isotropic HFCCs in MHz Computed for the Ground State of Alkali Atoms^a^

atom	wave function	RAS1	RAS2	RAS3	|**A**|
^7^Li	4c-HF				288.2
	CASSCF(1,1)		1 × s		282.3
	CASSCF(3,10)		4 × s, 2 × p		367.9
	CASSCF(3,15)		4 × s, 2 × p, 1 × d		367.9
	CASSCF(3,14)		5 × s, 3 × p		395.7
	RASSCF(3,29)	1 × s	1 × s	6 × s, 7 × p	392.8
	**Experimental**				**401.7**
^23^Na	4c-HF				633.4
	CASSCF(1,1)		1 × s		639.2
	CASSCF(9,13)		2 × s, 2 × p, 1 × d		724.6
	CASSCF(9,14)		3 × s, 2 × p, 1 × d		794.2
	CASSCF(11,15)		4 × s, 2 × p, 1 × d		801.9
	RASSCF(11,35)	2 × s, 1 × p	1 × s	6 × s, 6 × p, 1 × d	820.9
	RASSCF(11,40)	2 × s, 1 × p	1 × s	6 × s, 6 × p, 2 × d	839.6
	**Experimental**				**885.8**
^39^K	4c-HF				151.0
	CASSCF(1,1)		1 × s		152.9
	CASSCF(9,13)		2 × s, 2 × p, 1 × d		184.0
	CASSCF(9,14)		3 × s, 2 × p, 1 × d		197.1
	CASSCF(11,15)		4 × s, 2 × p, 1 × d		203.3
	CASSCF(11,16)		5 × s, 2 × p, 1 × d		210.7
	RASSCF(19,39)	3 × s, 2 × p	1 × s	6 × s, 6 × p, 1 × d	203.6
	**Experimental**				**230.8**
^85^Rb	4c-HF				666.9
	CASSCF(1,1)		1 × s		691.9
	CASSCF(9,13)		2 × s, 2 × p, 1 × d		831.2
	CASSCF(11,15)		4 × s, 2 × p, 1 × d		906.5
	RASSCF(19,26)	1 × s, 1 × p, 1 × d	1 × s	1 × s, 1 × p, 1 × d, 1 × f	859.1
	RASCI(37,54)	4 × s, 3 × p, 1 × d	1 × s	5 × s, 6 × p, 1 × d, 1 × f	908.8
	**Experimental**				**1011.9**
^133^Cs	4c-HF				1495.5
	CASSCF(1,1)		1 × s		1539.1
	CASSCF(9,13)		2 × s, 2 × p, 1 × d		1863.9
	CASSCF(9,14)		3 × s, 2 × p, 1 × d		2024.6
	CASSCF(11,15)		4 × s, 2 × p, 1 × d		2041.3
	RASCI(35,40)	5 × s, 4 × p	1 × s	5 × s, 4 × p, 1 × d	2083.2
	RASCI(35,47)	5 × s, 4 × p	1 × s	5 × s, 4 × p, 1 × d, 1 × f	2095.7
	**Experimental**				**2298.1**
^223^Fr	4c-HF				5518.0
	CASSCF(1,1)		1 × s		5240.1
	CASSCF(9,13)		2 × s, 2 × p, 1 × d		6277.0
	CASSCF(11,15)		4 × s, 2 × p, 1 × d		6685.7
	RASCI(43,57)	6 × s, 5 × p	1 × s	5 × s, 6 × p, 1 × d, 1 × f	6940.0
	**Experimental**				**7654.0**

a The RAS1, RAS2, and RAS3 columns indicate the number of atomic shells—separated by angular momentum—included in each subspace. Experimental HFCCs and four-component Hartree-Fock (4c-HF) HFCCs are reproduced from ref (22).

The balance of doubly occupied and virtual (unoccupied) orbitals in the CAS/RAS has a crucial influence on HFCC accuracy; it is not sufficient to augment a minimal active space with core orbitals, as appropriate virtual orbitals are needed to correlate them. A similar observation was made in a recent coupled-cluster study^22^ that emphasizes the need for high-energy unoccupied orbitals to correlate inner-core electrons. From our results, we deduce that radial correlation, introduced via virtual shells with the same angular momentum as the core–shells,^76^ has the most significant effect on HFCCs. Compare, for example, the HFCCs obtained from CASSCF(9,13) and CASSCF(9,14); the virtual s shell included in the latter leads to a 6–8% improvement in accuracy. For calculations that include orbital optimization (i.e., CASSCF or RASSCF), a good basic principle for active space selection is to include one radially correlating virtual shell for each doubly occupied shell in the active space. However, the exponential scaling of CASSCF severely restricts this strategy, and as such, the largest CAS selections reported herein include only one virtual shell of each angular momentum.

It is also interesting to analyze the influence of polarization basis functions. The inclusion of one virtual d shell in CASSCF affects the HFCCs of all alkali by 4–5% (except Li), suggesting an angular correlation^76^ effect related to the presence of core p electrons. Extrapolating, we postulate that, for core–shells with the highest angular momentum *l*, virtual shells with an angular momentum of at least *l* + 1 are needed to capture angular correlation. Note that, although previous theoretical work highlights the slow convergence of correlation energy with maximum angular momentum^77^—indicating that an accurate description of correlation likely requires much higher angular momenta—similar effects on HFCCs have only been explored for light atoms.^13,16^ Nevertheless, testing this hypothesis here is unfeasible given the computational limitations of CASSCF and RASSCF algorithms; we therefore limit our approach to include one virtual d shell for atoms Na–Fr and additionally one virtual f shell for Rb–Fr. Nonetheless, we come within 8–12% total relative error for these atoms.

**Figure 2:**
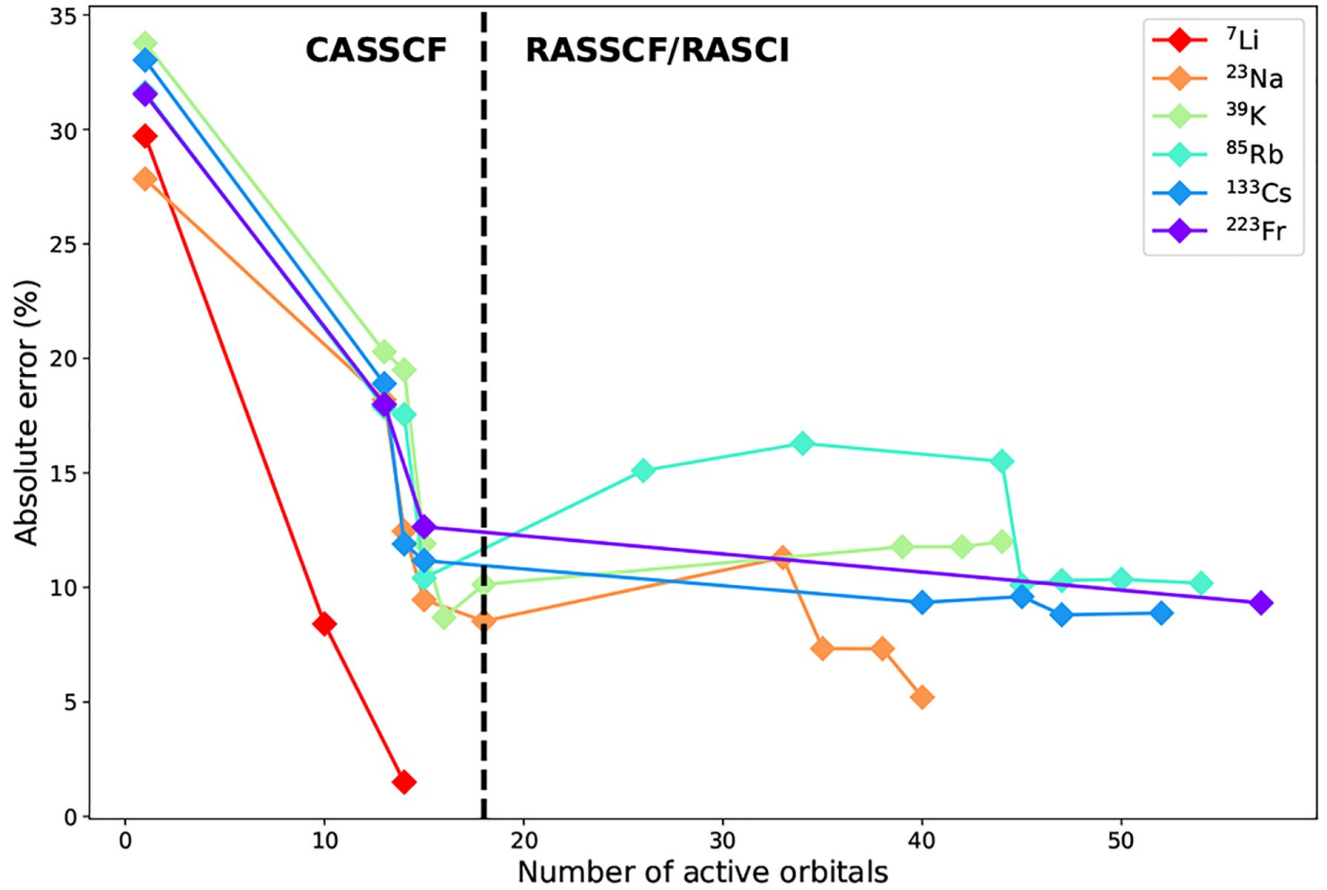
Evolution of HFCC absolute error with active space size for alkali atoms. Absolute errors are computed with respect to experimental data from ref (22).

Spin-dependent HFCCs are known to be particularly sensitive to correlation effects from inner-core electrons; to investigate this, we performed RASSCF and RASCI calculations with RAS1 subspaces spanning most of the core region. All RASCI calculations are performed with CASSCF(11,15)-optimized orbitals for consistency. Orbital decomposition analysis of RASCI results (Figure 1a) reveals a trend of decreasing HFC contribution with increasing angular momentum. Most s and p orbitals are strongly coupled by the HFC operator and make significant (>10 MHz) direct contributions to the HFCC. The influence of (virtual) polarization functions (4–5% increase in HFCC accuracy) is not reflected by the relatively insignificant contributions (<10^–3^ MHz) shown in the orbital decomposition diagram. Therefore, such functions have a predominantly indirect effect, displacing spin density from orbitals with significant HFC contributions. Occupied d and f shells show similarly small orbital decomposition contributions, and additionally, our calculations suggest their inclusion leads to insignificant variations in the computed HFCC. As such, we conclude that core d and f orbitals of alkali atoms can be safely left out of the RAS1 subspace for the purpose of HFCC determinations. We note that the error with respect to experimental HFCCs plateaus around 10% for RASCI (Figure 2), which could be a consequence of the restricted excitation level and/or the basis set size. These results are nevertheless very encouraging, considering the highly correlated nature of atomic systems, as well as the fact that our calculations explore a relatively limited parameter space (contracted basis sets, RAS1/RAS3 subspaces limited to no more than 35 orbitals).

Lastly, the issue of orbital optimization in RAS calculations bears discussion. The super-CI algorithm employed by OpenMolcas “folds” single excitations involving inactive and secondary orbitals into the active MO coefficients,^68^ thus capturing more correlation than a CI-only calculation with the same parameters. Theoretically, orbital optimization should allow for accurate HFCCs to be obtained with relatively little computational expense, since the active space needs only be large enough to capture the significant correlation contributions to HFC; indeed, this proves true for CASSCF; however, for RASSCF, the results are unpredictable. In the case of light alkali (Li–K), it was possible to run RASSCF calculations involving the entire core s and p manifold, together with polarization functions, which produced HFCCs in excellent agreement with experiment. On the other hand, this approach is prohibitive for heavier atoms—even if memory is not a limitation, orbital optimization is slow and prone to convergence issues. We therefore only employ RASCI for large active space calculations on Rb–Fr. RASSCF calculations involving fewer active orbitals were also attempted; however, the resulting HFCCs were on average less accurate than those from RASCI. It appears that the excitation level restriction severely hinders the efficiency of orbital optimization; stochastic CASSCF^78^ and DMRG-CASSCF methods^79^ are likely to provide more accurate HFCCs; however, due to additional uncertainty introduced by approximate CI solvers, such algorithms are not explored herein.

### 4.3 Coinage Metals

The treatment of HFC in coinage metals is very similar to that of the alkali metals, given they both have single-reference ^2^S_1/2_ ground states. A notable point of difference is the valence d shell which, unlike the core-like d shells in alkali atoms, imparts a significant contribution to the HFCC. Correlating the entire valence region is therefore not feasible with CASSCF, and in this case, RASSCF is the only option. We find that the benefits of a larger active space outweigh the limitations due to excitation level restrictions, leading to a net improvement in HFCC accuracy from CASSCF to RASSCF (Table 2). This contrasts with the behavior observed for the alkali metals, indicating that it is perhaps the nature of the correlated orbitals (valence vs core), rather than the CI approximation, that makes the biggest difference to theoretical HFCCs.

**Table 2:** Unsigned Isotropic HFCCs in MHz Computed for the Ground States of Cu, Ag, and Au^a^

atom	wave function	RAS1	RAS2	RAS3	|**A**|
^63^Cu	CASSCF(11,6)		1 × s, 1 × d		4137.3
	CASSCF(19,14)		3 × s, 2 × p, 1 × d		4604.9
	RASSCF(19,19)	1 × s, 1 × p, 1 × d	1 × s	1 × s, 1 × p, 1 × d	4611.3
	RASSCF(19,26)	1 × s, 1 × p, 1 × d	1 × s	1 × s, 1 × p, 1 × d, 1 × f	4831.4
	RASSCF(19,35)	1 × s, 1 × p, 1 × d	1 × s	1 × s, 1 × p, 1 × d, 1 × f, 1 × g	4813.7
	RASSCF(29,45)	3 × s, 2 × p, 1 × d	1 × s	3 × s, 2 × p, 1 × d, 1 × f, 1 × g	5004.8
	RASCI(29,50)	3 × s, 2 × p, 1 × d	1 × s	5 × s, 6 × p, 1 × d, 1 × f	5036.5
	**Experimental**				**5866.9**
^107^Ag	CASSCF(11,6)		1 × s, 1 × d		1325.1
	CASSCF(19,14)		3 × s, 2 × p, 1 × d		1455.1
	RASSCF(19,19)	1 × s, 1 × p, 1 × d	1 × s	1 × s, 1 × p, 1 × d	1481.1
	RASSCF(19,26)	1 × s, 1 × p, 1 × d	1 × s	1 × s, 1 × p, 1 × d, 1 × f	1594.6
	RASCI(37,54)	4 × s, 3 × p, 1 × d	1 × s	5 × s, 6 × p, 1 × d, 1 × f	1620.6
	RASCI(47,59)	4 × s, 3 × p, 2 × d	1 × s	5 × s, 6 × p, 1 × d, 1 × f	1619.4
	**Experimental**				**1712.5**
^197^Au	CASSCF(11,6)		1 × s, 1 × d		2362.1
	CASSCF(19,14)		3 × s, 2 × p, 1 × d		2536.0
	RASSCF(19,19)	1 × s, 1 × p, 1 × d	1 × s	1 × s, 1 × p, 1 × d	2608.3
	RASSCF(19,26)	1 × s, 1 × p, 1 × d	1 × s	1 × s, 1 × p, 1 × d, 1 × f	2733.6
	RASCI(45,54)	5 × s, 4 × p, 1 × d	1 × s	4 × s, 5 × p, 1 × d, 1 × f	2704.6
	**Experimental**				**3049.7**

a The RAS1, RAS2, and RAS3 columns indicate the number of atomic shells—separated by angular momentum—included in each subspace. Experimental HFCCs are reproduced from ref (75).

All RASCI calculations reported herein were performed using a RASSCF(19,26)-optimized orbital space for all coinage metals. Surprisingly, correlating most of the s and p manifold changes the initial RASSCF HFCCs very little; the largest variations are observed in calculations that correlate additional polarization shells. We observe improvements of 2–6% upon including one f shell in the orbital optimization step (cf. the RASCI results in Tables 2 and S2). Despite the significant challenges associated with these elements, Hyperion is still able to achieve relative errors of less than 15%.

Some notable differences between the coinage metals and the alkali are highlighted by the orbital decomposition analysis. Figure 1 shows orbital decomposition diagrams obtained from RASCI calculations on ^133^Cs and ^197^Au atoms; while both systems have a 6s^1^ ground configuration, their core configurations set them apart, which is reflected by the observed HFC. Compared to ^133^Cs, ^197^Au exhibits stronger coupling between d orbitals, as well as more significant contributions from the f polarization shell (however, the latter could be a consequence of including this shell in the orbital optimization step). Nevertheless, in both cases, the largest contributions to HFC are concentrated around orbitals 5s–7p, with additional non-negligible couplings involving the most diffuse s and p functions.

### 4.4 Groups VI-B (Cr) and VIII-B (Fe)

The hyperfine structure of transition metal (TM) atoms with partially filled d shells is by far the most challenging to model due to a number of competing factors. The orbital angular momentum couples to the nuclear spin through the PSO mechanism, and the resulting HFCC contribution is similar in magnitude to the spin-dependent FC+SD contribution (Figure S3). Both SOC and spin density must therefore be modeled accurately. On the one hand, the RASSI approach requires a sufficient number of spin-adapted states in order to represent the SOC states accurately, where the number of optimized roots corresponds to the lowest-energy Russell–Saunders (*LS*) terms (Table 3). Additional *LS* terms were included for ^101^Ru, ^183^W, and ^189^Os to obtain a converged ordering of SO energies at the minimal CASSCF-SO level.

**Table 3:** Number of Spin-Adapted Roots Optimized for Each Spin *S* and Corresponding *LS* Terms

atom	number of roots	*LS* terms
^53^Cr	1 (*S* = 3), 6 (*S* = 2)	^7^S, ^5^D, ^5^S
^95^ Mo	1 (*S* = 3), 6 (*S* = 2)	^7^S, ^5^D, ^5^S
^183^W	1 (*S* = 3), 5 (*S* = 2), 3 (*S* = 1)	^7^S, ^5^D, ^3^P
^57^Fe	12 (*S* = 2)	^5^D, ^5^F
^101^Ru	7 (*S* = 2), 7 (*S* = 1)	^5^F, ^3^F
^189^Os	12 (*S* = 2), 10 (*S* = 1)	^5^D, ^5^F, ^3^P, ^3^F

**Figure 3:**
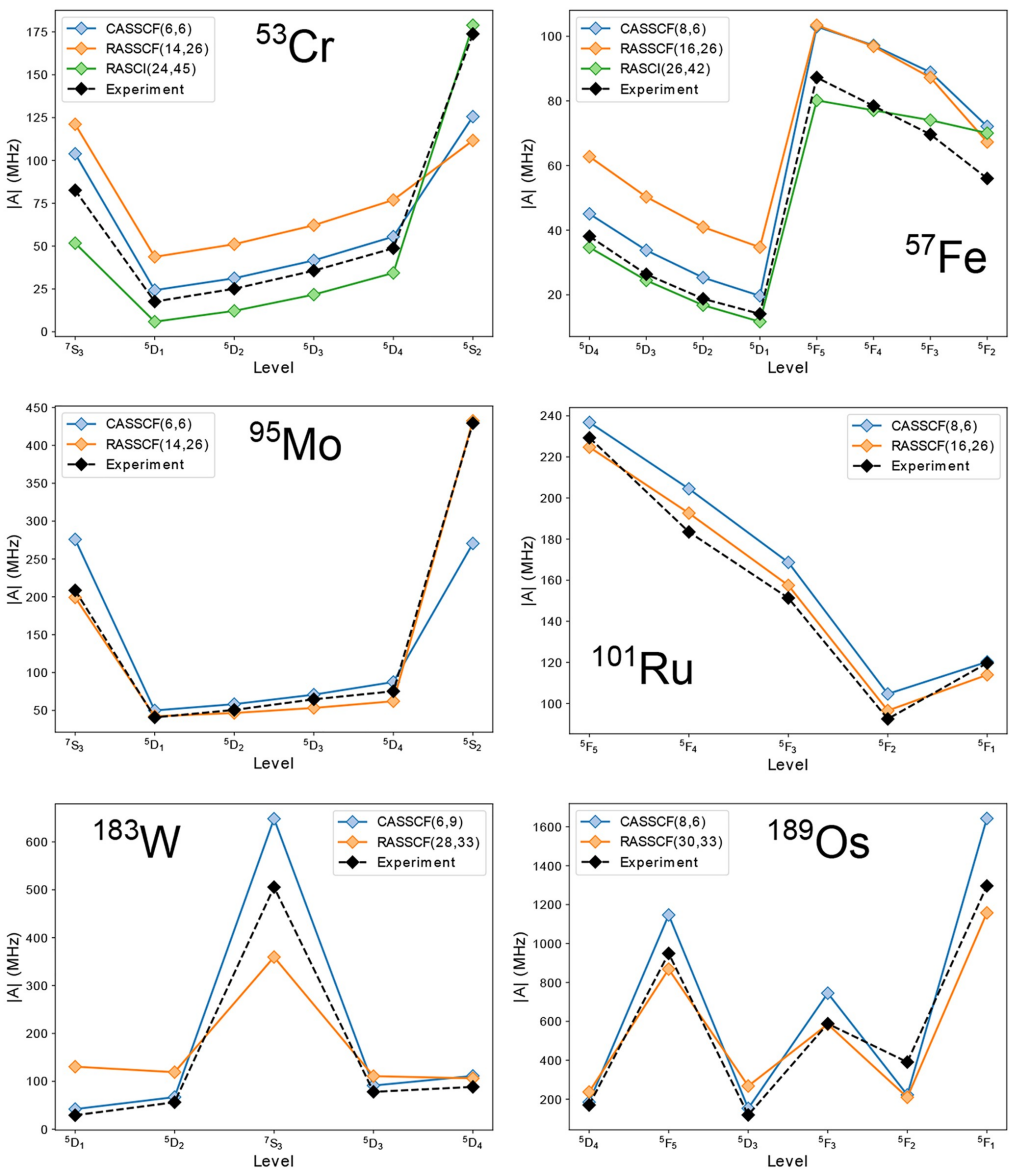
Unsigned HFCCs computed by Hyperion for selected energy levels of Cr group and Fe group atoms. Experimental HFCCs are reproduced from ref (80) (^53^Cr), (81) (^95^Mo, ^183^W), (82) and (83) (^57^Fe), (84) (^101^Ru), and (85) (^189^Os).

On the other hand, the electronic states of TM atoms exhibit significant mixing between *n*d^*N*^(*n* + 1)s^2^ and *n*d^*N*+1^(*n* + 1)s^1^ configurations; these have competing influences on the form of the valence s orbital, as optimized singly occupied s functions are usually more radially expanded than doubly occupied s functions.^76^ The  dependence of the HFC operator amplifies such differences, leading to computed HFCCs that are very sensitive to variations in the CI expansion. This proves particularly problematic when CASSCF/RASSCF orbitals are averaged over states dominated by different configurations, such as the low-lying ^5^D (3d^6^4s^2^) and ^5^F (3d^7^4s^1^) terms of ^57^Fe.^82,83^ Note that CASSCF(8,6)-SO HFCCs for ^101^Ru are in much better agreement with experiment than ^57^Fe HFCCs (Figure 3); this is because the ^101^Ru ^5^F term is sufficiently energetically separated and can be modeled without requiring additional quintet roots in the spin-adapted CASSCF step. Similar state-averaging effects are observed in the quintet levels of ^53^Cr and ^95^Mo.

Although relatively small in magnitude, the spin-dependent part of TM HFCCs depends on SP and higher-order correlation, similar to the spin-only HFCCs of alkali and coinage metals. Unlike the ^2^S_1/2_ systems, however, the expanded RAS2 subspace precludes large RAS optimizations. Therefore, calculations that correlate the entire core region were only feasible with RASCI for 3d TMs; however, this still gave very good results (Figure 3). RASCI-SO HFCCs computed for ^57^Fe are overall improved compared to CASSCF(8,6)-SO; meanwhile, RASCI-SO HFCCs for all ^53^Cr energy levels except ^5^S_2_ are slightly worse than those obtained from CASSCF-SO. We note that the RASCI-SO energies determined for ^53^Cr do not match the ordering observed experimentally and that the wrong ground state is predicted, indicating inaccuracies in the electronic wave function that are reflected in the calculated HFCCs. RASSCF-SO calculations correlating the valence shells were performed for all six atoms, yielding HFCCs that are overall less accurate compared to CASSCF-SO for the 3d and 5d TMs. The improvement with RASSCF observed for ^95^Mo and ^101^Ru can be justified by their well separated ground terms, which prevents state contamination during the RASSCF optimization.

Overall, it appears that minimal CASSCF-SO provides the most balanced model for the hyperfine structure of TMs, with errors around 25%. Approaches that include more correlation effects can theoretically improve the accuracy of the FC+SD term; in practice, however, the state-averaging formalism employed in CASSCF-SO, combined with the number of optimized spin-free states—which are likely characterized by different dynamical correlation effects—worsen the quality of the SO-coupled wave functions,^86,87^ which propagates to the computed HFCCs.

### 4.5 Lanthanides

The hyperfine structure of lanthanide (Ln) atoms is dominated by the PSO term, with previous work suggesting minor contributions from core polarization.^88^ As the 6s orbital and the 4f manifold are energetically well separated, CI effects between the two are negligible and the HFC response is expected to arise predominantly from the 4f shell. Hence, we compute the HFCCs of multiple levels in 4f^*N*^6s^2^ Ln atoms using a minimal CASSCF(*N*,7)-SO, with the number of optimized roots (Table 4) selected using a similar approach to section 4.4.

**Table 4:** CASSCF(*N*,7)-SO-Optimized *LS* Terms for Each Ln Atom

atom	*LS* terms
^141^Pr	^4^I
^143^Nd	^5^I
^147^Pm	^6^H
^147^Sm	^7^F, ^5^D
^151^Eu	^8^S
^159^Tb	^6^H, ^4^I
^161^Dy	^5^I
^165^Ho	^4^I
^167^Er	^3^H, ^3^F, ^1^G
^169^Tm	^2^F

Predicted HFCCs (Table 5) are in remarkable agreement with experiment overall.^89^ The poorest agreement is observed for ^151^Eu and ^165^Ho; the former has a spin-only septet ground state, which only exhibits spin-dependent HFC (no PSO contribution), and hence requires a more sophisticated treatment of correlation. Inaccuracies in the latter could also be due to missing CI effects, as indicated in previous work,^89^ but increasing the active space with CASSCF or RASSCF methods had no appreciable impact on the calculated HFCCs here.

**Table 5:** Unsigned Isotropic HFCCs in MHz Determined for Ln Atoms^a^

atom	level	|**A**^FC+SD^|	|**A**^PSO^|	|**A**^tot^|	|**A**^expt^|
^141^Pr	^4^I_9/2_	28.6	933.9	962.5	926.2
	^4^I_11/2_	13.9	759.4	773.4	730.4
	^4^I_13/2_	0.4	654.8	655.1	613.2
	^4^I_15/2_	13.3	587.0	573.8	541.6
^143^Nd	^5^I_4_	6.2	207.5	201.3	195.7
	^5^I_5_	3.8	163.1	159.3	153.7
	^5^I_6_	1.7	137.7	135.9	130.6
	^5^I_7_	0.3	121.8	122.1	117.6
	^5^I_8_	2.4	111.2	113.6	110.5
^147^Pm	^6^H_7/2_	42.0	472.3	430.4	447.1
^147^Sm	^7^F_1_	34.8	70.4	35.6	33.5
	^7^F_2_	28.6	70.4	41.8	41.2
	^7^F_3_	20.4	70.4	50.0	50.2
	^7^F_4_	11.2	70.4	59.2	59.7
	^7^F_5_	1.7	70.4	68.7	69.1
	^7^F_6_	7.7	70.4	78.1	78.4
^151^Eu	^8^S_7/2_	1.8	0.0	1.8	20.1
^159^Tb	^6^H_15/2_	25.2	720.0	745.2	673.8
	^6^H_13/2_	2.8	773.4	770.7	682.9
	^6^H_11/2_	34.0	856.8	822.9	729.0
^161^Dy	^5^I_8_	2.7	124.9	127.6	116.2
	^5^I_7_	0.3	136.8	137.1	126.8
	^5^I_6_	1.9	154.6	152.7	139.6
	^5^I_5_	4.3	183.2	178.9	162.0
	^5^I_4_	6.9	233.2	226.2	205.2
^165^Ho	^4^I_15/2_	28.6	1253.7	1225.1	800.6
	^4^I_13/2_	0.7	1398.4	1399.0	937.2
	^4^I_11/2_	29.8	1621.9	1651.7	1035.1
	^4^I_9/2_	61.5	1994.5	2056.0	1137.7
^167^Er	^3^H_6_	9.2	136.2	127.0	120.5
	^3^F_4_	16.8	147.1	130.4	121.8
	^3^H_5_	4.7	158.0	162.7	159.5
	^3^H_4_	5.2	168.4	173.6	173.4
	^3^F_3_	4.6	149.8	145.2	143.5
	^3^F_2_	14.4	217.9	203.5	167.1
^169^Tm	^2^F_7/2_	48.4	433.0	384.7	374.1
	^2^F_5/2_	115.8	577.4	693.2	704.6

a Experimental HFCCs are reproduced from ref (89).

## 5 Conclusions

We have presented a new computational package, Hyperion, that enables the evaluation of relativistic picture-change-corrected magnetic resonance parameters from CASSCF-SO and RASSCF-SO wave functions, along with a new orbital decomposition method to assist in choosing appropriate active spaces for HFCC calculations. We used this code to study the hyperfine structure of alkali metal, transition metal, and lanthanide atoms in order to understand the range of applicability afforded by this approach. Our best predicted HFCCs are within 10% accuracy for alkali, 15% for coinage metals, and 20% for lanthanides (although the vast majority of Ln HFCCs deviate less than 10% from the experimental value). The hyperfine structure of group VI-B (Cr) and group VIII-B (Fe) transition metals proved to be the most challenging to model; however, despite the larger percentage errors, we obtain theoretical HFCCs that closely follow experimentally observed trends. Based on these results, we devised a number of guidelines for modeling HFC in systems exhibiting important correlation effects, strong SOC, or a combination of both. In future work, these guidelines will be refined by using Hyperion to study HFC in molecular systems.

## Supporting Information Available

Discussion of active space selection criteria using orbital decomposition analysis and additional Hyperion results for alkali and transition metal atoms (PDF)

## Notes

The Hyperion code is available as free open-source software at https://gitlab.com/chilton-group/hyperion.

## References

[ref1] Mar G. N. L. NMR of Paramagnetic Molecules; Academic Press: 1973; pp 85–126.

[ref2] Chipman D. M. The spin polarization model for hyperfine coupling constants. Theoretica Chimica Acta 1992, 82, 93–115. 10.1007/BF01113132.

[ref3] Formanuik A.; Ariciu A.-M. M.; Ortu F.; Beekmeyer R.; Kerridge A.; Tuna F.; McInnes E. J. L.; Mills D. P. Actinide covalency measured by pulsed electron paramagnetic resonance spectroscopy. Nat. Chem. 2017, 9, 578–583. 10.1038/nchem.2692. 28537586

[ref4] Chen J.; Hu C.; Stanton J. F.; Hill S.; Cheng H. P.; Zhang X. G. Decoherence in Molecular Electron Spin Qubits: Insights from Quantum Many-Body Simulations. J. Phys. Chem. Lett. 2020, 11, 2074–2078. 10.1021/acs.jpclett.0c00193. 32097549

[ref5] Martin B.; Autschbach J. Temperature dependence of contact and dipolar NMR chemical shifts in paramagnetic molecules. J. Chem. Phys. 2015, 142, 054108 10.1063/1.4906318. 25662637

[ref6] Parker D.; Suturina E. A.; Kuprov I.; Chilton N. F. How the Ligand Field in Lanthanide Coordination Complexes Determines Magnetic Susceptibility Anisotropy, Paramagnetic NMR Shift, and Relaxation Behavior. Acc. Chem. Res. 2020, 53, 1520–1534. 10.1021/acs.accounts.0c00275. 32667187 PMC7467575

[ref7] Bennati M. EPR Interactions - Hyperfine Couplings. eMagRes. 2017, 6, 271–282. 10.1002/9780470034590.emrstm1503.

[ref8] Hermosilla L.; Calle P.; García De La Vega J. M.; Sieiro C. Density functional theory predictions of isotropic hyperfine coupling constants. J. Chem. Phys. 2005, 109, 1114–1124. 10.1021/jp0466901. 16833420

[ref9] Kossmann S.; Kirchner B.; Neese F. Performance of modern density functional theory for the prediction of hyperfine structure: Meta-GGA and double hybrid functionals. Mol. Phys. 2007, 105, 2049–2071. 10.1080/00268970701604655.

[ref10] Engels B. Calculation of NMR and EPR Parameters: Theory and Applications; Wiley-VCH: 2004; Chapter 30, pp 483–492.

[ref11] Witwicki M.; Walencik P. K.; Jezierska J. How accurate is density functional theory in predicting spin density? An insight from the prediction of hyperfine coupling constants. J. Mol. Model. 2020, 26, 10 10.1007/s00894-019-4268-0. 31834497

[ref12] Engels B.; Peyerimhoff S. D.; Davidson E. R. Calculation of hyperfine coupling constants an ab initio MRD-CI study for nitrogen to analyse the effects of basis sets and CI parameters. Mol. Phys. 1987, 62, 109–127. 10.1080/00268978700102091.

[ref13] Engels B.; Peyerimhoff S. D. The hyperfine coupling constants of the X^3^Σ^–^ states of NH influence of polarization functions and configuration space on the description of spin polarization. Mol. Phys. 1989, 67, 583–600. 10.1080/00268978900101301.

[ref14] Engels B. Estimation of the influence of the configurations neglected within truncated multi-reference CI wavefunctions on molecular properties. Chem. Phys. Lett. 1991, 179, 398–404. 10.1016/0009-2614(91)85173-T.

[ref15] Feller D.; Davidson E. R. A multireference CI determination of the isotropic hyperfine constants for first row atoms B-F. J. Chem. Phys. 1988, 88, 7580–7587. 10.1063/1.454322.

[ref16] Bauschlicher C. W.; Langhoff S. R.; Partridge H.; Chong D. P. Theoretical study of the nitrogen atom hyperfine coupling constant. J. Chem. Phys. 1988, 89, 2985–2992. 10.1063/1.455004.

[ref17] Bauschlicher C. W. Theoretical study of the nitrogen-atom hyperfine coupling constant. II. J. Chem. Phys. 1990, 92, 518–521. 10.1063/1.458600.

[ref18] Carmichael I. Ab initio calculation of the hyperfine coupling constants in B_2_. J. Chem. Phys. 1989, 91, 1072–1078. 10.1063/1.457179.

[ref19] Munzarova M. L.; Kubacek P.; Kaupp M. Mechanisms of EPR hyperfine coupling in transition metal complexes. J. Am. Chem. Soc. 2000, 122, 11900–11913. 10.1021/ja002062v.

[ref20] Kutzelnigg W. Origin and meaning of the Fermi contact interaction. Theoretica Chimica Acta 1988, 73, 173–200. 10.1007/BF00528203.

[ref21] Arratia-Perez R.; Case D. A. Relativistic effects on molecular hyperfine interactions: Application to XeF and CsO. J. Chem. Phys. 1983, 79.4939 10.1063/1.445587

[ref22] Talukdar K.; Sasmal S.; Nayak M. K.; Vaval N.; Pal S. Correlation trends in the magnetic hyperfine structure of atoms: A relativistic coupled-cluster case study. Phys. Rev. A 2018, 98, 022507 10.1103/PhysRevA.98.022507.

[ref23] Quiney H. M.; Belanzoni P. Relativistic calculation of hyperfine and electron spin resonance parameters in diatomic molecules. Chem. Phys. Lett. 2002, 353, 253–258. 10.1016/S0009-2614(01)01483-X.

[ref24] Gohr S.; Hrobárik P.; Repiský M.; Komorovský S.; Ruud K.; Kaupp M. Four-Component Relativistic Density Functional Theory Calculations of EPR g - And Hyperfine-Coupling Tensors Using Hybrid Functionals: Validation on Transition-Metal Complexes with Large Tensor Anisotropies and Higher-Order Spin-Orbit Effects. J. Phys. Chem. A 2015, 119, 12892–12905. 10.1021/acs.jpca.5b10996. 26636191

[ref25] Barysz M.; Sadlej A. J. Expectation values of operators in approximate two-component relativistic theories. Theor. Chem. Acc. 1997, 97, 260–270. 10.1007/s002140050260.

[ref26] Saue T. Relativistic Hamiltonians for chemistry: A primer. ChemPhysChem 2011, 12, 3077–3094. 10.1002/cphc.201100682. 22076930

[ref27] Liu W. Handbook of Relativistic Quantum Chemistry; Springer Nature: 2016.

[ref28] Van Lenthe E.; Van Der Avoird A.; Wormer P. E. Density functional calculations of molecular hyperfine interactions in the zero order regular approximation for relativistic effects. J. Chem. Phys. 1998, 108, 4783–4796. 10.1063/1.475889.

[ref29] Belanzoni P.; van Lenthe E.; Baerends E. J. An evaluation of the density functional approach in the zero order regular approximation for relativistic effects: Magnetic interactions in small metal compounds. J. Chem. Phys. 2001, 114, 4421–4433. 10.1063/1.1345509.

[ref30] Aquino F.; Pritchard B.; Autschbach J. Scalar relativistic computations and localized orbital analyses of nuclear hyperfine coupling and paramagnetic NMR chemical shifts. J. Chem. Theory Comput. 2012, 8, 598–609. 10.1021/ct2008507. 26596608

[ref31] Verma P.; Autschbach J. Relativistic density functional calculations of hyperfine coupling with variational versus perturbational treatment of spin-orbit coupling. J. Chem. Theory Comput. 2013, 9, 1932–1948. 10.1021/ct301114z. 26583544

[ref32] Autschbach J. The accuracy of hyperfine integrals in relativistic NMR computations based on the zeroth-order regular approximation. Theor. Chem. Acc. 2004, 112, 52–57. 10.1007/s00214-003-0561-0.

[ref33] Autschbach J. The role of the exchange-correlation response kernel and scaling corrections in relativistic density functional nuclear magnetic shielding calculations with the zeroth-order regular approximation. Mol. Phys. 2013, 111, 2544–2554. 10.1080/00268976.2013.796415.

[ref34] Autschbach J. Relativistic Methods for Chemists; Springer: Dordrecht, The Netherlands, 2010; pp 521–598.

[ref35] Malkin I.; Malkina O. L.; Malkin V. G.; Kaupp M. Scalar relativistic calculations of hyperfine coupling tensors using the Douglas-Kroll-Hess method. Chem. Phys. Lett. 2004, 396, 268–276. 10.1016/j.cplett.2004.08.037. 17028696

[ref36] Malkin E.; Malkin I.; Malkina O. L.; Malkin V. G.; Kaupp M. Scalar relativistic calculations of hyperfine coupling tensors using the Douglas-Kroll-Hess method with a finite-size nucleus model. Phys. Chem. Chem. Phys. 2006, 8, 4079–4085. 10.1039/B607044B. 17028696

[ref37] Nguyen Lan T.; Kurashige Y.; Yanai T. Scalar Relativistic Calculations of Hyperfine Coupling Constants Using Ab Initio Density Matrix Renormalization Group Method in Combination with Third-Order Douglas-Kroll-Hess Transformation: Case Studies on 4d Transition Metals. J. Chem. Theory Comput. 2015, 11, 73–81. 10.1021/ct5007778. 26574205

[ref38] Filatov M.; Cremer D. Relativistically corrected hyperfine structure constants calculated with the regular approximation applied to correlation corrected ab initio theory. J. Chem. Phys. 2004, 121, 5618–5622. 10.1063/1.1785772. 15366984

[ref39] Autschbach J. Relativistic Effects on Electron-Nucleus Hyperfine Coupling Studied with an Exact 2-Component (X2C) Hamiltonian. J. Chem. Theory Comput. 2017, 13, 710–718. 10.1021/acs.jctc.6b01014. 27973772

[ref40] Wodyński A.; Kaupp M. Density Functional Calculations of Electron Paramagnetic Resonance g - And Hyperfine-Coupling Tensors Using the Exact Two-Component (X2C) Transformation and Efficient Approximations to the Two-Electron Spin-Orbit Terms. J. Phys. Chem. A 2019, 123, 5660–5672. 10.1021/acs.jpca.9b03979. 31184482

[ref41] Feng R.; Duignan T. J.; Autschbach J. Electron-Nucleus Hyperfine Coupling Calculated from Restricted Active Space Wavefunctions and an Exact Two-Component Hamiltonian. J. Chem. Theory Comput. 2021, 17, 255–268. 10.1021/acs.jctc.0c01005. 33385321

[ref42] Filatov M.; Zou W.; Cremer D. Analytic calculation of isotropic hyperfine structure constants using the normalized elimination of the small component formalism. J. Phys. Chem. A 2012, 116, 3481–3486. 10.1021/jp301224u. 22424301

[ref43] Ishikawa Y.; Binning R. C.; Sando K. M. Dirac-Fock discrete-basis calculations on the beryllium atom. Chem. Phys. Lett. 1983, 101, 111–114. 10.1016/0009-2614(83)80314-5.

[ref44] Stanton R. E.; Havriliak S. Kinetic balance: A partial solution to the problem of variational safety in Dirac calculations. J. Chem. Phys. 1984, 81, 1910 10.1063/1.447865.

[ref45] Dyall K. G.; Fægri K. Kinetic balance and variational bounds failure in the solution of the Dirac equation in a finite Gaussian basis set. Chem. Phys. Lett. 1990, 174, 25–32. 10.1016/0009-2614(90)85321-3.

[ref46] Sun Q.; Liu W.; Kutzelnigg W. Comparison of restricted, unrestricted, inverse, and dual kinetic balances for four-component relativistic calculations. Theor. Chem. Acc. 2011, 129, 423–436. 10.1007/s00214-010-0876-6.

[ref47] Dyall K. G. An exact separation of the spin-free and spin-dependent terms of the Dirac-Coulomb-Breit Hamiltonian. J. Chem. Phys. 1994, 100, 2118 10.1063/1.466508.

[ref48] Dyall K. G. Introduction to relativistic quantum chemistry; Oxford University Press: New York, 2007.

[ref49] Kutzelnigg W.; Liu W. Quasirelativistic theory equivalent to fully relativistic theory. J. Chem. Phys. 2005, 123, 241102 10.1063/1.2137315. 16396527

[ref50] Kutzelnigg W.; Liu W. Quasirelativistic theory I. Theory in terms of a quasi-relativistic operator. Mol. Phys. 2006, 104, 2225–2240. 10.1080/00268970600662481.

[ref51] Liu W.; Kutzelnigg W. Quasirelativistic theory. II. Theory at matrix level. J. Chem. Phys. 2007, 126, 114107 10.1063/1.2710258. 17381196

[ref52] Liu W.; Peng D. Exact two-component Hamiltonians revisited. J. Chem. Phys. 2009, 131, 031104 10.1063/1.3159445. 19624172

[ref53] Neese F. Quantum chemistry and EPR parameters. eMagRes. 2017, 6, 1–22. 10.1002/9780470034590.emrstm1505.

[ref54] Arbuznikov A. V.; Vaara J.; Kaupp M. Relativistic spin-orbit effects on hyperfine coupling tensors by density-functional theory. J. Chem. Phys. 2004, 120, 2127–2139. 10.1063/1.1636720. 15268351

[ref55] Hennum A. C.; Klopper W.; Helgaker T. Direct perturbation theory of magnetic properties and relativistic corrections for the point nuclear and Gaussian nuclear models. J. Chem. Phys. 2001, 115, 7356–7363. 10.1063/1.1405009.

[ref56] Malmqvist P. Å.; Roos B. O.; Schimmelpfennig B. The restricted active space (RAS) state interaction approach with spin-orbit coupling. Chem. Phys. Lett. 2002, 357, 230–240. 10.1016/S0009-2614(02)00498-0.

[ref57] Chibotaru L. F. Advances in Chemical Physics; John Wiley & Sons, Ltd: 2013; pp 397–519.

[ref58] Bolvin H. An alternative approach to the g-matrix: Theory and applications. ChemPhysChem 2006, 7, 1575–1589. 10.1002/cphc.200600051. 16810728

[ref59] Boguslawski K.; Tecmer P.; Legeza Ö.; Reiher M. Entanglement Measures for Single- and Multireference Correlation. J. Phys. Chem. Lett. 2012, 3, 3129–3135. 10.1021/jz301319v. 26296018

[ref60] Aquilante F.; Ferré N.; Autschbach J.; Conti I.; Baiardi A.; Vico L. D.; Battaglia S.; Veniamin A.; Ernst D.; Norell J.; Lindh R.; Delcey M.; Galván I. F.; Freitag L.; Garavelli M.; Gong X.; Knecht S.; Nenov A.; Lundberg M.; Schapiro I.; Odelius M.; Phung Q. M.; Ungur L.; Segatta F.; Olivucci M.; Seijo L.; Pedersen T. B.; Pedrazagonzález L.; Segarra-martí J.; Pierloot K.; Vacher M.; Reiher M.; Valentini A.; Veryazov V.; Conti I.; Galván I. F.; Freitag L. Modern quantum chemistry with [Open ] Molcas Modern quantum chemistry with [Open ] Molcas. J. Chem. Phys. 2020, 152, 214117 10.1063/5.0004835. 32505150

[ref61] Sun Q. Libcint: An efficient general integral library for Gaussian basis functions. J. Comput. Chem. 2015, 36, 1664–1671. 10.1002/jcc.23981. 26123808

[ref62] Roos B. O.; Lindh R.; Malmqvist P. Å.; Veryazov V.; Widmark P. O. Main Group Atoms and Dimers Studied with a New Relativistic ANO Basis Set. J. Phys. Chem. A 2004, 108, 2851–2858. 10.1021/jp031064+.

[ref63] Roos B. O.; Veryazov V.; Widmark P. O. Relativistic atomic natural orbital type basis sets for the alkaline and alkaline-earth atoms applied to the ground-state potentials for the corresponding dimers. Theor. Chem. Acc. 2004, 111, 345–351. 10.1007/s00214-003-0537-0.

[ref64] Roos B. O.; Lindh R.; Malmqvist P. Å.; Veryazov V.; Widmark P. O. New relativistic ANO basis sets for actinide atoms. Chem. Phys. Lett. 2005, 409, 295–299. 10.1016/j.cplett.2005.05.011. 16834004

[ref65] Roos B. O.; Lindh R.; Malmqvist P. Å.; Veryazov V.; Widmark P. O. New relativistic ANO basis sets for transition metal atoms. J. Phys. Chem. A 2005, 109, 6575–6579. 10.1021/jp0581126. 16834004

[ref66] Roos B. O.; Lindh R.; Malmqvist P. Å.; Veryazov V.; Widmark P. O.; Borin A. C. New relativistic atomic natural orbital basis sets for lanthanide atoms with applications to the Ce diatom and LuF3. J. Phys. Chem. A 2008, 112, 11431–11435. 10.1021/jp803213j. 18928264

[ref67] Sharkas K.; Pritchard B.; Autschbach J. Effects from spin-orbit coupling on electron-nucleus hyperfine coupling calculated at the restricted active space level for Kramers doublets. J. Chem. Theory Comput. 2015, 11, 538–549. 10.1021/ct500988h. 26580911

[ref68] Roos B. O. The complete active space SCF method in a Fock-matrix-based super-CI formulation. Int. J. Quantum Chem. 1980, 18, 175–189. 10.1002/qua.560180822.

[ref69] Malmqvist P. Å.; Rendell A.; Roos B. O. The restricted active space self-consistent-field method, implemented with a split graph unitary group approach. J. Phys. Chem. 1990, 94, 5477–5482. 10.1021/j100377a011.

[ref70] Li Z.; Xiao Y.; Liu W. On the spin separation of algebraic two-component relativistic Hamiltonians. J. Chem. Phys. 2012, 137, 154114 10.1063/1.4758987. 23083155

[ref71] Heß B. A.; Marian C. M.; Wahlgren U.; Gropen O. A mean-field spin-orbit method applicable to correlated wavefunctions. Chem. Phys. Lett. 1996, 251, 365–371. 10.1016/0009-2614(96)00119-4.

[ref72] Kollmar H.; Staemmler V. Violation of Hundas rule by spin polarization in molecules. Theoretica Chimica Acta 1978, 48, 223–239. 10.1007/BF00549021.

[ref73] Giner E.; Tenti L.; Angeli C.; Ferré N. Computation of the Isotropic Hyperfine Coupling Constant: Efficiency and Insights from a New Approach Based on Wave Function Theory. J. Chem. Theory Comput. 2017, 13, 475–487. 10.1021/acs.jctc.6b00827. 28094936

[ref74] Tterlikkis L.; Mahanti S.; Das T. Theoretical Analysis of the Hyperfine Structure of Alkali Atoms. Phys. Rev. 1968, 176, 10–19. 10.1103/PhysRev.176.10.

[ref75] Lindgren I.; Rosén A. Case Studies in Atomic Physics; Elsevier: 1975; pp 197–298.

[ref76] Helgaker T.; Taylor P. R. Modern Electronic Structure Theory; World Scientific: 1995; pp 725–856.

[ref77] Kutzelnigg W. r12-Dependent terms in the wave function as closed sums of partial wave amplitudes for large l. Theoretica Chimica Acta 1985, 68, 445–469. 10.1007/BF00527669.

[ref78] Li Manni G.; Smart S. D.; Alavi A. Combining the Complete Active Space Self-Consistent Field Method and the Full Configuration Interaction Quantum Monte Carlo within a Super-CI Framework, with Application to Challenging Metal-Porphyrins. J. Chem. Theory Comput. 2016, 12, 1245–1258. 10.1021/acs.jctc.5b01190. 26808894

[ref79] Battaglia S.; Keller S.; Knecht S. Efficient Relativistic Density-Matrix Renormalization Group Implementation in a Matrix-Product Formulation. J. Chem. Theory Comput. 2018, 14, 2353–2369. 10.1021/acs.jctc.7b01065. 29558618

[ref80] Jarosz A.; Stefańska D.; Elantkowska M.; Ruczkowski J.; Buczek A.; Furmann B.; Głowacki P.; Krzykowski A.; Pitkowski; Stachowska E.; Dembczyński J. High precision investigations of the hyperfine structure of metastable levels in a chromium atom. Journal of Physics B: Atomic, Molecular and Optical Physics 2007, 40, 2785–2797. 10.1088/0953-4075/40/13/019.

[ref81] Büttgenbach S. Hyperfine Structure in 4d- and 5d-Shell Atoms; Springer-Verlag: Berlin, Heidelberg, 1982.

[ref82] Childs W. J.; Goodman L. S. Hyperfine Interactions and the Magnetic Fields Due to Core Polarization in Fe^57^. Phys. Rev. 1966, 148, 74–78. 10.1103/PhysRev.148.74.

[ref83] Dembczyński J.; Ertmer W.; Johann U.; Stinner P. High precision measurements of the hyperfine structure of seven metastable atomic states of ^57^Fe by laser-Rf double-resonance. Zeitschrift für Physik A Atoms and Nuclei 1980, 294, 313–317. 10.1007/BF01434138.

[ref84] Forest D. H.; Powis R. A.; Cochrane E. C.; Griffith J. A.; Tungate G. High resolution laser spectroscopy of naturally occurring ruthenium isotopes. Journal of Physics G: Nuclear and Particle Physics 2014, 41, 025106 10.1088/0954-3899/41/2/025106.

[ref85] Kröger S.; Başar G.; Baier A.; Guthöhrlein G. H. Hyperfine Structure and Isotope Shift of Osmium I. Phys. Scr. 2002, 65, 56–68. 10.1238/Physica.Regular.065a00056.

[ref86] Ganyushin D.; Neese F. A fully variational spin-orbit coupled complete active space self-consistent field approach: Application to electron paramagnetic resonance g-tensors. J. Chem. Phys. 2013, 138, 104113 10.1063/1.4793736. 23514471

[ref87] Seed J. A.; Birnoschi L.; Lu E.; Tuna F.; Wooles A. J.; Chilton N. F.; Liddle S. T. Anomalous magnetism of uranium(IV)-oxo and -imido complexes reveals unusual doubly degenerate electronic ground states. Chem. 2021, 7, 1666–1680. 10.1016/j.chempr.2021.05.001.

[ref88] Childs W. J.; Crowsswhite H.; Goodman L. S.; Pfeufer V. Hyperfine structure of 4f^*N*^6s^2^ configurations in ^159^Tb, ^161,163^Dy, and ^169^Tm. Journal of the Optical Society of America B 1984, 1, 22–29. 10.1364/JOSAB.1.000022.

[ref89] Cheng K. T.; Childs W. J. Ab initio calculation of 4f^*N*^6s^2^ hyperfine structure in neutral rare-earth atoms. Phys. Rev. A 1985, 31, 2775–2784. 10.1103/PhysRevA.31.2775. 9895831

