# Supplementary material for: Hyperion: A New Computational Tool for Relativistic Ab Initio Hyperfine Coupling
Source: J Chem Theory Comput. 2022 Jul 1;18(8):4719–32. doi: 10.1021/acs.jctc.2c00257 (PMC9367016; doi:10.1021/acs.jctc.2c00257)
Supplement: Supplementary file 1 — ct2c00257_si_001.pdf [file ct2c00257_si_001.pdf]

Supporting Information: Hyperion: a New Computational Tool for Relativistic Ab Initio Hyperfine Coupling

Letitia Birnoschi* and Nicholas F. Chilton*

Department of Chemistry, The University of Manchester, Oxford Road, Manchester, M13 9PL, United Kingdom

E-mail: letitia.birnoschi@manchester.ac.uk; nicholas.chilton@manchester.ac.uk

S1 Investigation of active space selection using orbital decomposition analysis

To investigate the influence of various orbital optimisation strategies on the computed HFCCs, we choose two small test systems, the ^{7}Li atom and the ^{14}N atom, and represent both using an ANO-RCC...8s7p basis set (containing 8 s functions and 7 sets of p functions). We have already shown that active spaces comprising only s and p shells are sufficient to obtain ^{7}Li HFCCs in excellent agreement with experiment (Table 1, main text); meanwhile, previous work[S1–S3] suggests that our chosen basis set is insufficient to achieve quantitative agreement with the experimental ^{14}N HFCC, $A = 10.4509$ MHz.[S1] Nevertheless, in this case we are interested not in the accuracy of the computed HFCC, but in the HFC orbital decomposition analysis.

We aim to model HFC in the full orbital space, which is achievable via RASCI(3,29) for the chosen test systems. Figures S1 and S2 show orbital decomposition diagrams for the $^{2}S_{1/2}$ ground state of ^{7}Li and for the $^{4}S_{3/2}$ ground state of ^{14}N, respectively; all diagrams are obtained from RASCI(3,29) calculations using CASSCF- and RASSCF-optimised orbitals. It is immediately apparent that, as the number of optimised orbitals increases, the diagrams become more sparse and the HFC response is focused into a smaller subset of the orbital space. Surprisingly, CASSCF and RASSCF orbitals obtained using the same active space selection give rise to extremely similar diagrams – the same pairwise couplings are observed in both cases, with only a few small changes in magnitude. The most noticeable difference is the coupling between optimised orbitals, which appears to be slightly stronger when RASSCF is used. The larger HFCCs observed for calculations using RASSCF orbitals are likely a result of this stronger coupling, especially as the variations in HFCC are small.

These observations suggest that, within the multiconfigurational electronic structure frame-

work, large active space optimisations of the orbital space result in the most effective representation of HFC. Compared to active space size, the constraints introduced by a RAS ansatz appear to have a minimal effect on the HFC orbital decomposition and the resulting HFCC. Of course, these conclusions are based on a limited number of small systems and might have limited applicability; further investigation is necessary to establish the validity of such trends in larger, more complex systems.

Finally, it is worth highlighting that orbital energy is not a good indicator of the contribution to HFC. In both ^{7}Li and ^{14}N, high-energy diffuse functions couple most strongly with the SOMO and the other valence orbitals, while "mid-range" orbitals (e.g. 2p-dominated functions in ^{7}Li, 5p-dominated functions in ^{14}N) have much smaller contributions. In the context of theoretical HFCC determinations, this is not a new observation; Feller and Davidson remarked, in a 1988 study of second-period atomic HFCCs,[S4] that "[...] *the choice of energy as the selection criterion is probably far from optimal for properties other than energy. However, when an entire group of properties is desired (including the energy) it may be as good a choice as any other.*" In *ab initio* studies that target HFCC accuracy, it is therefore essential to explore orbital selection criteria that are not energy-based; this is particularly relevant given the recent developments around automated active space selection methodologies.[S5–S7]

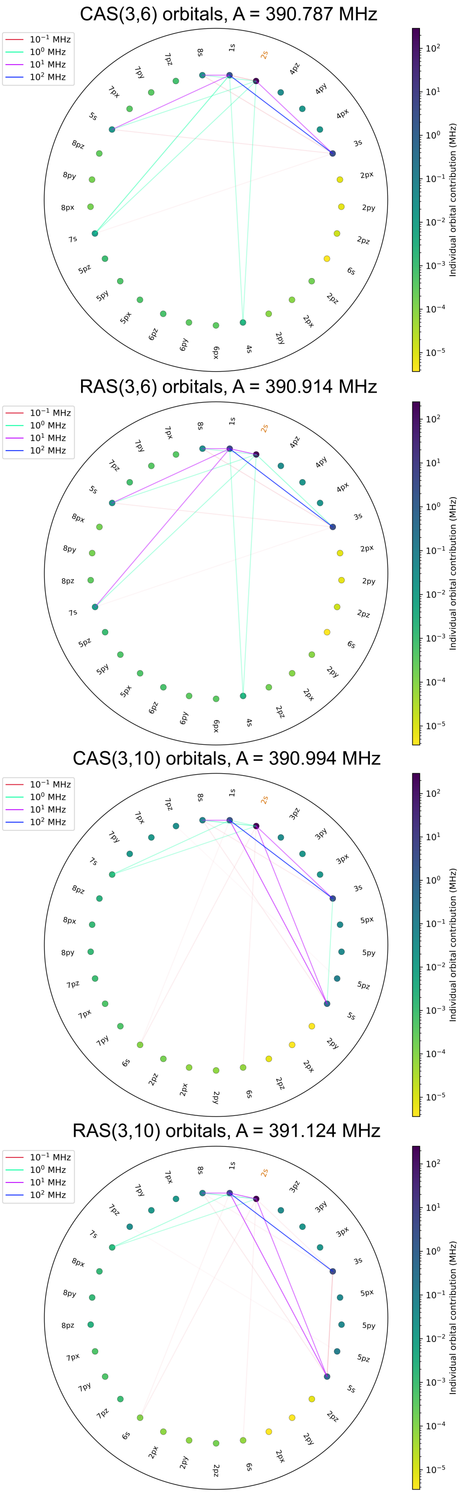

Figure S1: [7]Li HFCCs and HFC orbital decomposition diagrams determined from RASCI(3,29) wavefunctions using different CASSCF/RASSCF-optimised orbitals. Orbitals are labelled according to the dominant AO basis function contribution. Orange labels correspond to RAS2 orbitals.

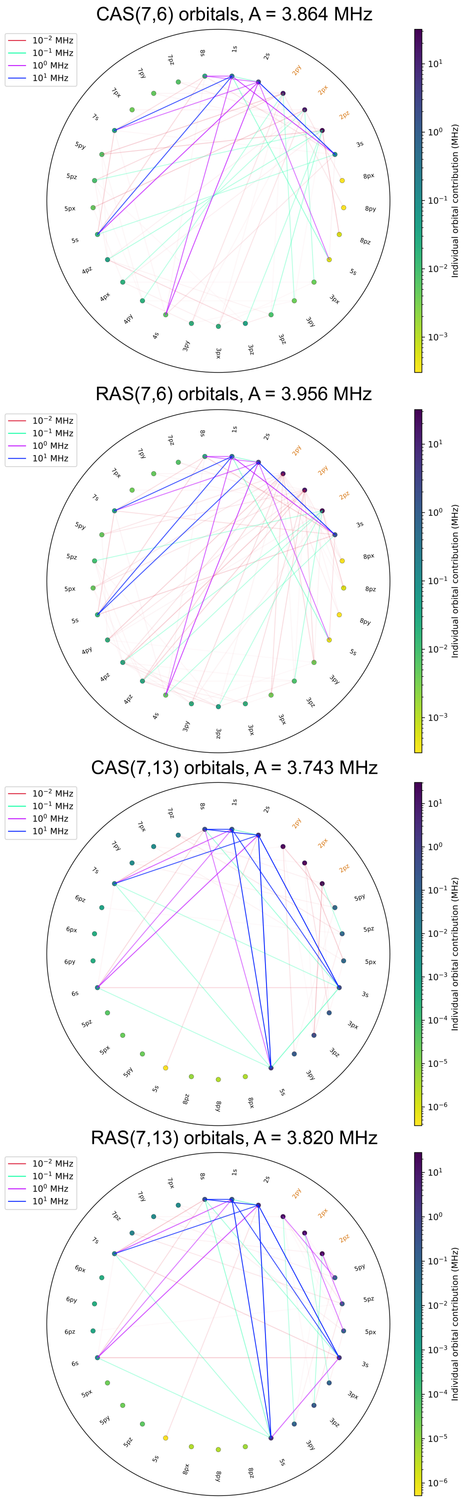

Figure S2: ^{14}N HFCCs and HFC orbital decomposition diagrams determined from RASCI(3,29) wavefunctions using different CASSCF/RASSCF-optimised orbitals. Orbitals are labelled according to the dominant AO basis function contribution. Orange labels correspond to RAS2 orbitals.

S2 Additional results

S2.1 Alkali

Table S1: Isotropic HFCCs (unsigned) in MHz computed for the ground state of alkali atoms. The RAS1, RAS2 and RAS3 columns indicate the number of atomic shells – separated by angular momentum – included in each subspace. Experimental HFCCs are reproduced from reference S8.

Atom	Wavefunction	RAS1	RAS2	RAS3	|A|
^{7}Li	CASSCF(3,14)	-	8×s, 2×p	-	378.1
	**Experimental**				**401.7**
^{23}Na	CASSCF(11,14)	-	5×s, 3×p	-	775.4
	CASSCF(11,18)	-	4×s, 3×p, 1×d	-	810.2
	RASSCF(11,33)	2×s, 1×p	1×s	6×s, 7×p	785.7
	RASSCF(11,38)	2×s, 1×p	1×s	6×s, 7×p, 1×d	820.8
	**Experimental**				**885.8**
^{39}K	CASSCF(11,14)	-	5×s, 3×p	-	185.8
	CASSCF(11,18)	-	4×s, 3×p, 1×d	-	207.4
	RASSCF(19,42)	3×s, 2×p	1×s	6×s, 7×p, 1×d	203.6
	RASSCF(19,44)	3×s, 2×p	1×s	6×s, 6×p, 2×d	203.1
	**Experimental**				**230.8**
^{85}Rb	CASSCF(11,14)	-	5×s, 3×p	-	834.3
	RASSCF(27,30)	2×s, 2×p, 1×d	1×s	1×s, 1×p, 1×d, 1×f	789.2
	RASSCF(27,34)	2×s, 2×p, 1×d	1×s	2×s, 2×p, 1×d, 1×f	847.0
	RASSCF(37,44)	4×s, 3×p, 1×d	1×s	4×s, 3×p, 1×d, 1×f	855.0
	RASCI(27,45)	4×s, 3×p	1×s	5×s, 7×p, 1×d	909.6
	RASCI(37,50)	4×s, 3×p, 1×d	1×s	5×s, 7×p, 1×d	907.2
	RASCI(37,47)	4×s, 3×p, 1×d	1×s	5×s, 6×p, 1×d	907.6
	**Experimental**				**1011.9**
^{133}Cs	RASCI(35,47)	5×s, 4×p	1×s	6×s, 6×p, 1×d	2070.9
	RASCI(43,51)	4×s, 4×p, 1×d	1×s	6×s, 6×p, 1×d	2066.2
	RASCI(45,52)	5×s, 4×p, 1×d	1×s	6×s, 6×p, 1×d	2066.8
	RASCI(45,45)	5×s, 4×p, 1×d	1×s	5×s, 4×p, 1×d	2077.4
	RASCI(45,52)	5×s, 4×p, 1×d	1×s	5×s, 4×p, 1×d, 1×f	2093.9
	**Experimental**				**2298.1**

S2.2 Coinage metals

Table S2: Isotropic HFCCs (unsigned) in MHz computed via RASCI using RASSCF(19,19)-optimised orbitals. The RAS1, RAS2 and RAS3 columns indicate the number of atomic shells – separated by angular momentum – included in each subspace. Experimental HFCCs are reproduced from reference S9.

Atom	Wavefunction	RAS1	RAS2	RAS3	|A|
^{63}Cu	RASCI(29,47)	3×s, 2×p, 1×d	1×s	6×s, 7×p, 1×d	4793.4
	RASCI(29,48)	3×s, 2×p, 1×d	1×s	6×s, 5×p, 1×d, 1×f	4908.1
	RASCI(29,49)	3×s, 2×p, 1×d	1×s	6×s, 6×p, 2×d	4826.8
	**Experimental**				**5866.9**
^{107}Ag	RASCI(47,52)	4×s, 3×p, 2×d	1×s	5×s, 6×p, 1×d	1495.4
	RASCI(47,57)	4×s, 3×p, 2×d	1×s	5×s, 6×p, 2×d	1505.1
	RASCI(47,59)	4×s, 3×p, 2×d	1×s	5×s, 6×p, 1×d, 1×f	1512.7
	**Experimental**				**1712.5**
^{197}Au	RASCI(45,47)	5×s, 4×p, 1×d	1×s	4×s, 5×p, 1×d	2598.0
	RASCI(45,54)	5×s, 4×p, 1×d	1×s	4×s, 5×p, 1×d, 1×f	2645.9
	**Experimental**				**3049.7**

S2.3 Groups VI-B (Cr) and VIII-B (Fe)

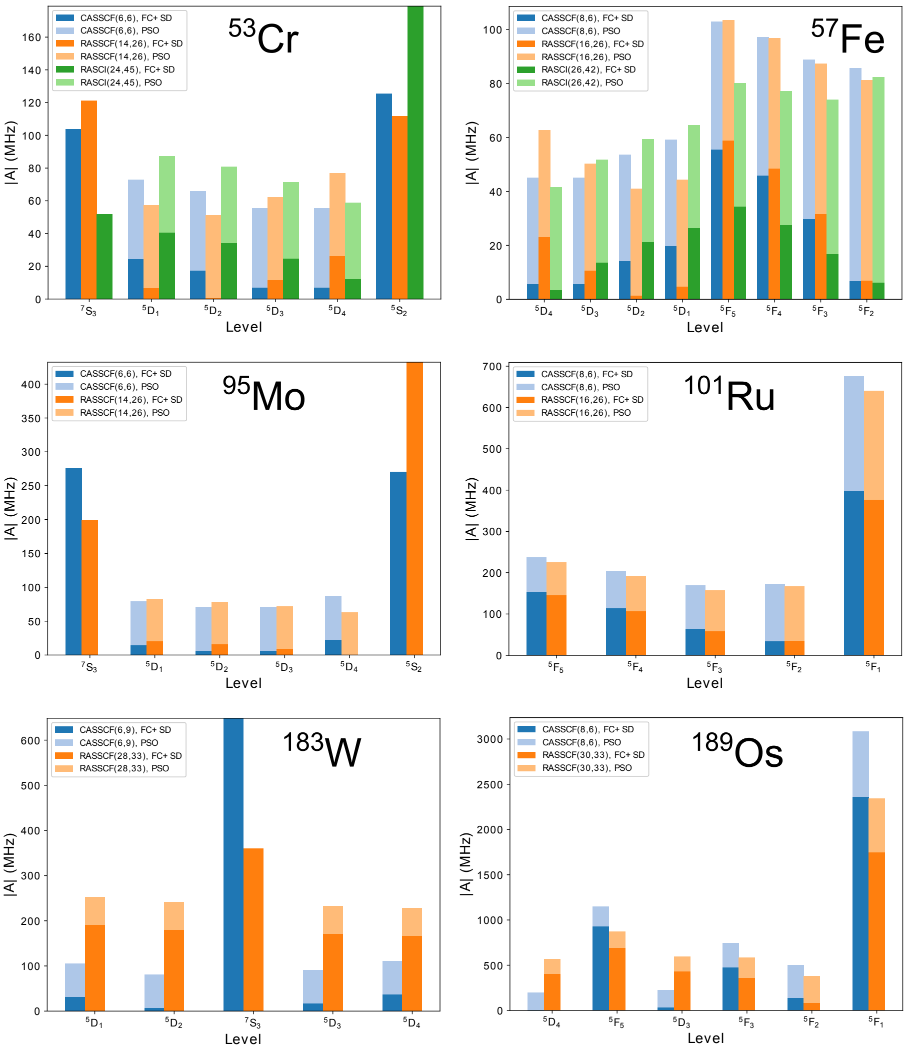

Figure S3: Spin-dependent (FC+SD) and spin-independent (PSO) unsigned HFCCs computed by Hyperion for selected energy levels of Cr group and Fe group atoms.

References

(S1) Engels, B.; Peyerimhoff, S. D.; Davidson, E. R. Calculation of hyperfine coupling constants an ab initio MRD-CI study for nitrogen to analyse the effects of basis sets and CI parameters. *Mol. Phys.* **1987**, *62*, 109–127.

(S2) Bauschlicher, C. W.; Langhoff, S. R.; Partridge, H.; Chong, D. P. Theoretical study of the nitrogen atom hyperfine coupling constant. *Journal of Chemical Physics* **1988**, *89*, 2985–2992.

(S3) Bauschlicher, C. W. Theoretical study of the nitrogen-atom hyperfine coupling constant. II. *Journal of Chemical Physics* **1990**, *92*, 518–521.

(S4) Feller, D.; Davidson, E. R. A multireference CI determination of the isotropic hyperfine constants for first row atoms B-F. *Journal of Chemical Physics* **1988**, *88*, 7580–7587.

(S5) Stein, C. J.; Reiher, M. Automated Identification of Relevant Frontier Orbitals for Chemical Compounds and Processes. *CHIMIA* **2017**, *71*, 170–176.

(S6) King, D.; Gagliardi, L. The Ranked-Orbital Approach to Selecting Active Spaces The Ranked-Orbital Approach to Selecting Active Spaces. *Journal of Chemical Theory and Computation* **2021**, *17*, 2817–2831.

(S7) Lei, Y.; Suo, B.; Liu, W. iCAS: Imposed Automatic Selection and Localization of Complete Active Spaces. *Journal of Chemical Theory and Computation* **2021**, *17*, 4846–4859.

(S8) Talukdar, K.; Sasmal, S.; Nayak, M. K.; Vaval, N.; Pal, S. Correlation trends in the magnetic hyperfine structure of atoms: A relativistic coupled-cluster case study. *Physical Review A* **2018**, *98*, 1–8.

(S9) Lindgren, I.; Rosén, A. *Case Studies in Atomic Physics*; Elsevier, 1975; pp 197–298.
